# Aluminum sulfate significantly reduces the skin test response to common allergens in sensitized patients

**DOI:** 10.1186/1476-7961-4-1

**Published:** 2006-02-14

**Authors:** C Steven Smith, Scott A Smith, Thomas J Grier, David E Justus

**Affiliations:** 1 Private practice, Fellow of the College and Academy of Asthma, Allergy and Immunology, Louisville, KY, USA; 2 Department of Pediatrics, University of Louisville School of Medicine, Louisville, KY, USA; 3 Department of Microbiology and Immunology, University of Louisville School of Medicine, Louisville, KY, USA; 4 Research and Development Laboratory, Greer Laboratories Inc., P.O. Box 800, Lenoir, NC, USA

## Abstract

**Background:**

Avoidance of allergens is still recommended as the first and best way to prevent allergic illnesses and their comorbid diseases. Despite a variety of attempts there has been very limited success in the area of environmental control of allergic disease. Our objective was to identify a non-invasive, non-pharmacological method to reduce indoor allergen loads in atopic persons' homes and public environments. We employed a novel *in vivo *approach to examine the possibility of using aluminum sulfate to control environmental allergens.

**Methods:**

Fifty skin test reactive patients were simultaneously skin tested with conventional test materials and the actions of the protein/glycoprotein modifier, aluminum sulfate. Common allergens, dog, cat, dust mite, Alternaria, and cockroach were used in the study.

**Results:**

Skin test reactivity was significantly reduced by the modifier aluminum sulfate. Our studies demonstrate that the effects of histamine were not affected by the presence of aluminum sulfate. In fact, skin test reactivity was reduced independent of whether aluminum sulfate was present in the allergen test material or removed prior to testing, indicating that the allergens had in some way been inactivated.

**Conclusion:**

Aluminum sulfate was found to reduce the *in vivo *allergic reaction cascade induced by skin testing with common allergens. The exact mechanism is not clear but appears to involve the alteration of IgE-binding epitopes on the allergen. Our results indicate that it may be possible to diminish the allergenicity of an environment by application of the active agent aluminum sulfate, thus producing environmental control without complete removal of the allergen.

## Background

The various clinical manifestations of type 1 hypersensitivities and their resultant comorbid illnesses are well known. A common feature shared by these is the mechanism by which they are induced, driven by cytokines produced by Th2 lymphocytes, resulting in IgE antibody production. Antigen specific IgE antibodies cause multiple preformed mediators to be released from mast cells and blood basophils. These preformed cytokines interact with their receptors on target cells inducing a cascade of reactions with late phase mediator formation, leading to sustained symptoms [1]. Consequently, anything that effectively interferes with or blocks the release of these mediators is of considerable interest in the prevention of allergic disease. Currently, methods employed for this purpose include pharmacological therapy, immunotherapy and avoidance of allergen(s). Although these applications have been of clinical benefit, they have not significantly lowered the number of new allergy cases and in particular asthmatics, numbering 16 million or 7.5% of the U.S. adult population [2]. This has been due to the difficulty reducing or preventing exposure to multiple ubiquitous environmental allergens [3]. Thus, any method(s) that effectively eliminates or greatly reduces these allergens from areas of exposure would have far reaching health benefits, reducing the mortality and morbidity burden of atopic individuals [4].

Although various attempts have been made to control environment allergens, most have met only limited success [5,6]. Chemicals such as tannic acid and sodium hypochlorite have been reported to form complexes with environmental allergens reducing their ability to trigger allergic reactions [6-12]. Unfortunately, these substances produce undesirable side effects such as unacceptable damage to treated surfaces. Acaricides applied to interior surfaces have proven to be unacceptable controls for dust mite allergens [13]. Sustained use of removal techniques for environmental control is met by most patients with resistance, especially where family pets are concerned [14]. Air filtration only picks up airborne allergens if they reach the filter before the patient, and constant vigilance of their function is imperative [15]. Filters which are too dense, result in poor air circulation and thereby reduced efficacy [16].

In this study, we employed a novel *in vivo *approach to examine the possibility of using aluminum sulfate (AS), to control environmental allergens and to inhibit allergic reactions. This chemical seemed to be a good candidate for this purpose because of its ability to bind to proteins [17], its long lasting residual effect, and its lack of toxicity [18,19]. Because of these functional properties, and the fact that allergens are for the most part composed of proteins or glycoproteins, we hypothesized that AS would interact with allergens. This interaction in turn would prevent them from triggering an allergic response by blocking their ability to bind with their specific IgE antibodies. This would eliminate a critical step in the allergic response cascade that generates the various clinical manifestations commonly associated with type 1 hypersensitivities. The results of this study demonstrate that AS can indeed inactivate a variety of allergens, blocking their ability to induce wheal and flare skin reactions in allergic individuals. This suggests that AS can be employed as an agent to interact with and inactivate environmental allergens.

## Methods

### Patient selection

Individuals were recruited from a large private allergy practice in Louisville, KY, between 1999 and 2005. The age range was from 10 to 66 yrs. These patients had clinical symptoms of rhinitis, asthma, conjunctivitis, chronic sinusitis or a combination of 2 or more of the manifestations of Type I hypersensitivity reactions. The protocol to be used in the study was explained in detail to the patients and they were given the option of participating in the study, informed consent was granted. If their prescribed routine allergy skin test produced a high level of reactivity to one of the selected test antigens they were included in the study groups. No volunteer was compensated monetarily or otherwise for his or her participation and no funding for the study came from outside sources.

### Aluminum sulfate solution preparations

Aluminum sulfate, Al_2_(SO_4_)_3_, (Sigma/Aldrich, St. Louis) was prepared by dissolving proper aliquots in sterile water. Using the same diluent as for the allergens (normal saline), 8.75% (ASα) and 34.2% (ASβ) solutions of the chemical were prepared. Prior to use, they were filtered using a micropore filter #4, placed in sterile containers, and stored at 4°C until used in skin test.

### Toxicity testing

Toxicity tests were performed using cultured human endothelial cells (obtained from ATCC, Rockville, MD; CRL 1730) and the trypan blue dye exclusion test to evaluate the safety of using AS in human applications [20]. Cells were incubated for 24 h with 1:10, 1:20, or 1:30 dilutions of ASβ, collected and examined microscopically for dye uptake. A hemocytometer was used for quantitation. Three separate tests were performed.

### Allergens used

Initial screening of patients involved only routine inhalant skin test with the following allergens: animal danders, cockroach, dust mite, mold spores and pollens of grasses, weeds and trees prevalent in the Ohio River Valley. They were obtained from Greer Labs (Lenoir, NC) and used at a standard prick test concentration. Those patients who produced high skin reactions (when compared to the diluent control, normal saline) to cat, dog, Alternaria, dust mites, cockroach or multiples there of were selected to undergo additional skin tests with these same allergens mixed with AS. AS-allergen preparations were created by mixing 0.1 ml ASα (8.75%) to 0.9 ml of the skin test dose allergen or 0.1 ml ASβ (34.2%) to 0.9 ml of the skin test dose allergen. No precipitate was observed upon mixing of the allergen and AS. All skin reactions were read 20 min after the prick test was applied.

### Skin test evaluation

The diameter of the patients' skin test responses to allergens or allergens mixed with AS were measured using skin test calipers and recorded in terms of size in mm of induration (wheal size) at the largest diameter. Clinical score: a conventional grading system of + to 4+ was also used as determined by these parameters: (+) = 3 to 5 mm wheal; (++) = 5 to 7 mm wheal; (+++) = 7 to 10 mm wheal; (++++) = 10 mm or greater wheal; erythema (E) and pseudopod (P) were also observed and recorded. P-values were determined using an unpaired two-tailed Student's t-test (< 0.05 was considered statistically significant).

### Histamine

Histamine base solution (1.8 mg/ml and 50% glycerol wt/vol), obtained from Allermed Labs (SanDiego, CA), was injected intradermally alone and mixed with AS (at a concentration of 0.9% and 9.0%). Controls included 0.9% NaCl, 0.4% phenol. After 20 min, lesion diameters were measured as before using skin test calipers. In some cases, reactive sites that had previously been injected with only histamine, received a second injection with just AS. As before, responses were read 20 min later.

### Sample dialysis

Lyophilized cat dander was purchased from Greer Labs (Lenoir, NC) and suspended in 100 microliters of sterile water to a concentration of 29 mg/ml. Next, 15 microliters was added to either 1 ml sterile water (sample 1) or 1 ml 9% AS (samples 2 & 3). Sample 1 was then dialyzed for 6 h against 1 liter of sterile water, sample 2 was dialyzed for 6 h against 1 liter of 9% AS, and sample 3 was dialyzed for 6 h against 1 liter of sterile water. Final allergen concentration was estimated to be approximately 50,000 BAU. Samples were then used for skin testing in three patients sensitized to cat allergen with inclusion of proper controls, sterile water as sample 4 and 9% AS solution as sample 5. The experiment was repeated using Centricon ultrafiltration devises (Millipore, 3K MW cutoff) as a different means to dialyze the samples. Dialysis was accomplished by addition of 2 ml of either 9% AS or water following complete concentration of the samples, this was repeated four times to ensure complete dialysis of the samples was accomplished.

## Results

### Aluminum sulfate exhibited no toxicity

A preliminary experiment was done to determine if AS exhibited any cellular toxicity. As previously noted, 1:10, 1:20, or 1:30 dilutions (diluted in normal saline) of ASβ were placed in established human endothelial cell cultures and incubated for 24 h with 5% CO_2 _at 37°C. Cells were collected and a trypan blue dye exclusion test was performed (see *Methods *section). The results indicate that 99.9% of the cells were still viable at the end of a 24 h exposure to the chemical, for the dilutions tested. It should also be noted here that no toxicity was observed *in vivo *using the skin prick test.

### Aluminum sulfate significantly reduces the allergen-induced skin prick response

**Figure 1:**
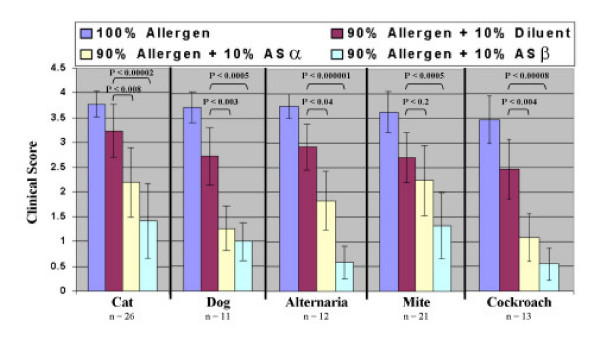
Selected patients were skin tested with allergens mixed with AS. Controls included allergens alone and saline. Means and standard deviations, as well as P-values, of data (clinical scores, see *Methods*) collected from the 50 patients are presented. P-values were determined using an unpaired two-tailed Student's t-test. A P-value of < 0.05 was considered statistically significant. Notice that AS markedly reduced skin test reactions to the allergens tested.

**Figure 2:**
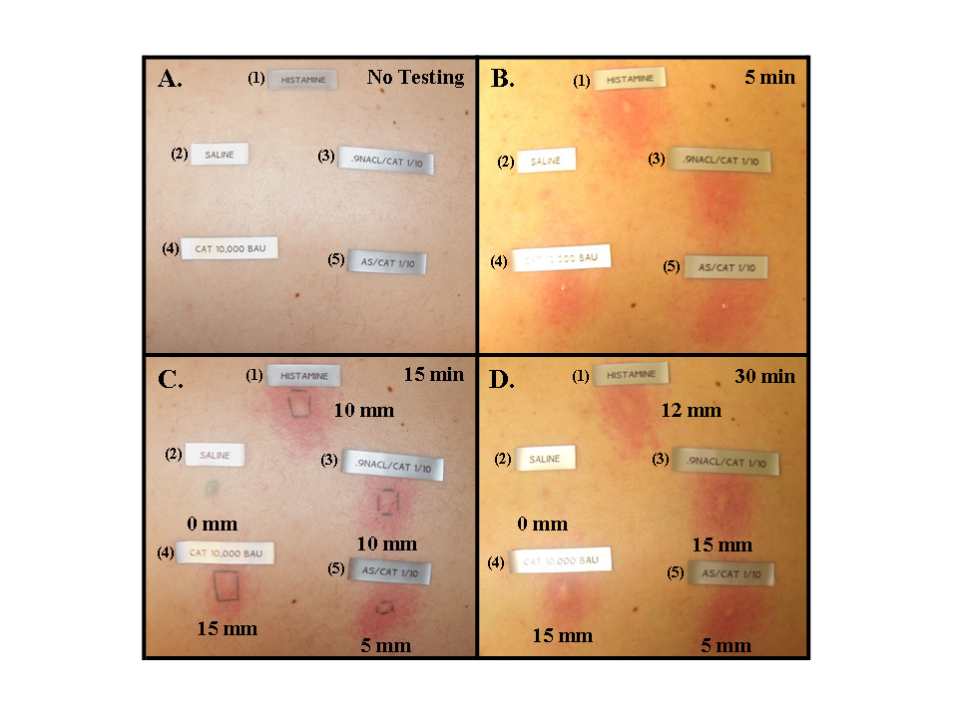
Wheal and flare responses in a patient skin tested with the cat allergen and cat allergen mixed with AS, as in Figure 1. Histamine and saline were included as controls. Reactions were read with no testing (A), 5 min (B), 15 min (C), and 30 min (D). Within each panel, skin prick responses are shown to: histamine (1), saline (2), 10% saline + 90% cat allergen (3), cat allergen (4), and 10% ASβ + 90% cat allergen (5). Wheals were measure by the aid of skin test calipers.

In this experiment, a group of 50 patients were used. They were selected for testing with the AS-allergen mixtures based on their level of sensitivity to routine allergy skin tests. For this purpose, only those allergens that had induced a +4 reaction (10 mm or greater) were mixed with AS for further skin testing. The allergens and their concentrations used are indicated in Table 1, and Figures 1 &2. A compilation of the results, recorded for each patient, appears in Table 1, while statistical analysis of this data is presented in Figure 1. As can be seen, in comparison to controls, significant decreases in skin reactivity (clinical score) to cat (P < 0.007), dog (P < 0.003), Alternaria (P < 0.03), and cockroach (P < 0.003) allergens occurred by the addition of AS. The given effect could be obtained by using either 8.75% or 34.2% dilutions of the AS. Amazingly, an even greater difference in patient skin test reactions were observed between sites receiving 90% allergen + 10% diluent ASα and those that received 90% allergen + 10% ASβ (in all cases P < 0.0005), demonstrating a dose response effect. From the data presented, it is quite clear that AS can interfere with mechanism(s) involved in type 1 hypersensitivity reactions. Clinically, the inhibitory effects of AS *in vivo *can more readily be seen by the photographic evidence presented in Figure 2.

**Table 1:** Patient skin test reactions to allergens and allergens mixed with AS. Summary of patient skin prick responses to dog, cat, mite, Alternaria, and cockroach allergens. Responses to 100% allergen, 90% allergen + 10% diluent, 90% allergen + 10% ASα, or 90% allergen + 10% ASβ were scored as follows: (+) = 3 to 5 mm wheal; (++) = 5 to 7 mm wheal; (+++) = 7 to 10 mm wheal; (++++) = 10 mm or greater wheal; E = erythema; P = pseudopod.

***Patient***	**Allergen Type**	**100% Allergen**	**90% Allergen + 10% Diluent**	**90% Allergen + 10% AS α**	**90% Allergen + 10% AS β**
**1**	Mite	++++EP (20 mm W)	++++EP (25 mm W)	++++EP (13 mm W)	++++EP (12 mm W)
**2**	Alternaria	+++ (7 mm W)	+++ (8 mm W)	+ (3 mm W)	0
**3**	Mite	++++	++ (5 mm W)	0 (2 mm E)	0
	Cockroach	++++	++ (6 mm W)	+ (3 mm W)	+ (4 mm W)
**4**	Cat	++++E (21 mm W)	++E (6 mm W)	0	0
	Mite	++++E (16 mm W)	+ (3 mm W)	+ (4 mm W)	0
**5**	Cockroach	++++E	++	++	0
**6**	Cockroach	++++E	++	++	0
**7**	Cat	+++	++	+	0
**8**	Cat	++++	++++	++++	0
	Mite	++++	++++	++++	0
**9**	Cat	++++ (20 mm W)	+ (3 mm W)	0	0
	Dog	++++ (20 mm W)	+ (3 mm W)	+ (3 mm W)	0
	Alternaria	+++E (7 mm W)	++ (4 mm W)	0	0
**10**	Cat	++++EP (10 mm W)	++++EP (9 mm W)	++ (5 mm W)	0
**11**	Cat	++++EP (10 mm W)	++E (5 mm W)	++E (5 mm W)	0 (1 mm W)
	Dog	++++EP (10 mm W)	++++EP (9 mm W)	++E (5 mm W)	++E (5 mm W)
	Mite	+++ (7 mm W)	+++ (7 mm W)	+ (3 mm W)	0 (1 mm W)
**12**	Cat	++++ (14 mm W)	++++ (14 mm W)	++++ (14 mm W)	+++ (6 mm W)
	Dog	++++ (10 mm W)	++++ (14 mm W)	+ (3 mm W)	+ (3 mm W)
	Alternaria	++++ (11 mm W)	++++ (10 mm W)	+++ (7 mm W)	0
	Mite	++++ (10 mm W)	+++ (7 mm W)	+++ (7 mm W)	+ (3 mm W)
	Cockroach	++++ (12 mm W)	++++ (10 mm W)	++ (6 mm W)	+ (4 mm W)
**13**	Cat	++++PE (15 mm W)	++++ (11 mm W)	++++ (12 mm W)	+ (3 mm W)
	Mite	++++E (19 mm W)	+++ (9 mm W)	+++ (9 mm W)	++ (5 mm W)
**14**	Cat	+++	+ (4 mm W)	++ (5 mm W)	+ (4 mm W)
	Alternaria	+++	+ (4 mm W)	+ (3 mm W)	0
	Mite	++++	++++ (13 mm W)	++++ (10 mm W)	++ (6 mm W)
**15**	Mite	+++ (8 mm W)	++ (5 mm W)	+ (3 mm W)	+ (3 mm W)
**16**	Cat	++++ (11 mm W)	++++ (18 mm W)	++++ (12 mm W)	++++ (16 mm W)
	Alternaria	++++ (21 mm W)	+++ (8 mm W)	++ (7 mm W)	+ (4 mm W)
	Mite	+++ (10 mm W)	++ (6 mm W)	++ (5 mm W)	+ (3 mm W)
**17**	Cat	++++EP (12 mm W)	++++EP (13 mm W)	+++E (8 mm W)	+++E (7 mm W)
	Mite	++++E (10 mm W)	++++E (10 mm W)	+++E (8 mm W)	+++E (6 mm W)
**18**	Mite	++++EP (15 mm W)	+++E (8 mm W)	++E (5 mm W)	++E (5 mm W)
**19**	Mite	++++E (10 mm W)	+++E (8 mm W)	++ (6 mm W)	+ (3 mm W)
**20**	Mite	++++EP (23 mm W)	++ (6 mm W)	++++ (11 mm W)	+ (3 mm W)
**21**	Cat	++++EP (11 mm W)	++++EP (11 mm W)	+++EP (8 mm W)	+++EP (8 mm W)
	Alternaria	++++	+++	+	+
	Mite	++++EP	+	+	+
**22**	Mite	++++E (9 mm W)	+++E (7 mm W)	++E (4 mm W)	+E (3 mm W)
**23**	Alternaria	++++	++ (3 mm W)	++ (3 mm W)	0
	Mite	+	+++E (7 mm W)	+++E (7 mm W)	0 (1 mm W)
**24**	Mite	+++E	+	0	0
	Cockroach	+++E	0	0	0
**25**	Mite	+++	+++	+	++
	Cockroach	++++E	+++E	+	+E
**26**	Cat	++++E (20 mm W)	++++E (13 mm W)	++++E (11 mm W)	++++E (19 mm W)
	Mite	++++ (10 mm W)	++E (5 mm W)	+E (4 mm W)	+E (3 mm W)
	Cockroach	++++ (15 mm W)	+++	0 E	0
**27**	Cat	++++ (10 mm W)	++++ (10 mm W)	0	0
	Mite	++++ (10 mm W)	++++ (10 mm W)	++++ (10 mm W)	++++ (10 mm W)
	Cockroach	++++ (10 mm W)	++++ (10 mm W)	0	+ (3 mm W)
**28**	Cockroach	++++ (10 mm W)	++++ (10 mm W)	+++ (7 mm W)	++ (6 mm W)
**29**	Cockroach	+E	+E	0	0
**30**	Cockroach	++++ (10 mm W)	+++ (7 mm W)	+ (3 mm W)	0
**31**	Cockroach	+++ (8 mm W)	++ (5 mm W)	+ (3 mm W)	+ (3 mm W)
**32**	Cat	++++ (10 mm W)	+++ (8 mm W)	+++ (8 mm W)	0
	Dog	++++ (10 mm W)	+++ (7 mm W)	+++ (7 mm W)	+ (5 mm W)
**33**	Cockroach	++ (6 mm W)	++ (6 mm W)	+ (3 mm W)	0
**34**	Cat	++++ (10 mm W)	++++ (14 mm W)	++ (6 mm W)	++ (7 mm W)
	Dog	++++ (10 mm W)	++++ (10 mm W)	0	0
**35**	Cat	++++ (18 mm W)	+++ (7 mm W)	+ (3 mm W)	+ (3 mm W)
	Dog	++++ (10 mm W)	++ (4 mm W)	+ (3 mm W)	++ (4 mm W)
**36**	Dog	++++E (12 mm W)	+++E (9 mm W)	++E (5 mm W)	+E (4 mm W)
**37**	Cat	++++E (10 mm W)	++++E (10 mm W)	+++ (7 mm W)	+ (4 mm W)
	Dog	++++EP (14 mm W)	++++EP (12 mm W)	0	0
**38**	Cat	++++ (11 mm W)	++++EP (13 mm W)	++ (6 mm W)	+ (3 mm W)
**39**	Alternaria	+++E (8 mm W)	+++E (9 mm W)	++ (5 mm W)	+ (4 mm W)
**40**	Alternaria	++++E (13 mm W)	++++E (11 mm W)	+++E (7 mm W)	0
**41**	Cat	+++ (8 mm W)	+++ (7 mm W)	++ (6 mm W)	++ (5 mm W)
**42**	Cat	++++E	++++E	+	++
**43**	Cat	+++ (8 mm W)	++ (6 mm W)	++ (5 mm W)	+ (4 mm W)
	Dog	++++ (11 mm W)	++ (5 mm W)	+ (4 mm W)	+ (4 mm W)
**44**	Alternaria	++++EP (12 mm W)	++++EP (12 mm W)	+++E (9 mm W)	++E (8 mm W)
**45**	Cat	++++EP	+++	++	+
	Alternaria	++++EP	++	++	+
**46**	Cat	++E	++E	0	+E
	Dog	++E (5 mm W)	++E (6 mm W)	++E (6 mm W)	+
**47**	Alternaria	++++E (10 mm W)	+++E (7 mm W)	++E (6 mm W)	+E (4 mm W)
**48**	Cat	++++E	++++E	+	0
**49**	Dog	+++E	+	+	++
**50**	Cat	++++EP (10 mm W)	++++EP (10 mm W)	+++E (8 mm W)	+++E (7 mm W)

### Aluminum sulfate does not block histamine effects

**Figure 3:**
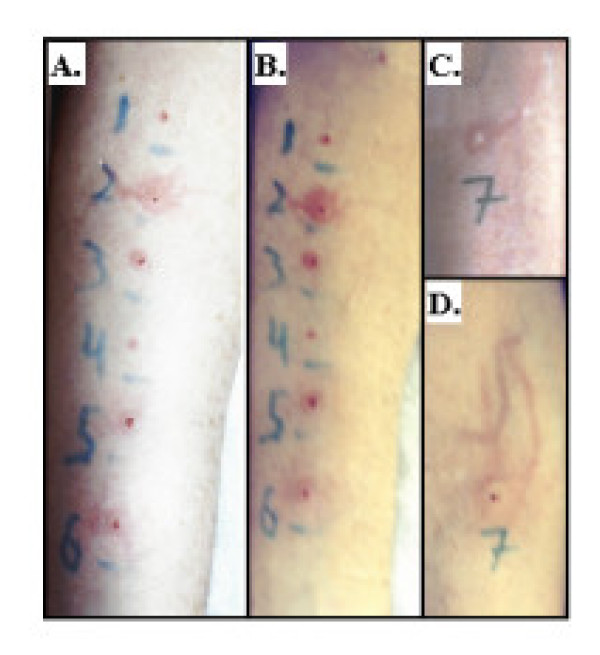
Aluminum sulfate does not block histamine. Skin reactions at 15 min are shown in panel (A). Skin reactions at 30 min are shown in panel (B). Within panels A and B, skin prick responses are shown to: saline (1), histamine (2), 0.9% AS (3), 9.0% AS (4), 0.9% AS + histamine (1.8 mg/ml) mixed at 1:10 (AS:histamine) (5), and 9.0% AS + histamine (1.8 mg/ml) mixed at 1:10 (AS:histamine) (6). Skin reaction to 0.1 cc AS ID at 15 min is shown in panel (C) (site 7). Skin reaction 15 min following overlay of site 7 in panel C with histamine (1.8 mg/ml) is shown in panel (D). Notice that AS did not inhibit the histamine induced skin reaction.

To determine if the observed AS induced reduction in STR possibly resulted from interference with histamine activity, a patient received injections of either histamine or histamine mixed with AS, as indicated (see *Methods *section). The results indicate that histamine induced skin reactions cannot be blocked by AS (Fig. 3). The size of the lesions for histamine (14 mm) and histamine-AS mixture (12 mm) was essentially the same. The experiment was repeated with the same results. This, in conjunction with the above finding, strongly suggests that AS exerts its effort by blocking allergen-IgE interactions.

### Exposure to aluminum sulfate alters the allergen

**Figure 4:**
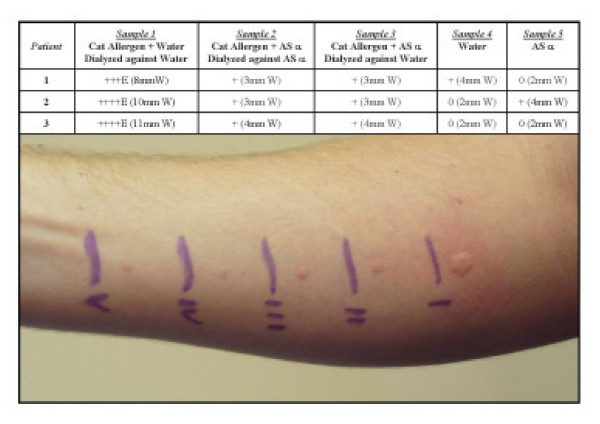
Exposure to aluminum sulfate alters the allergen. Skin test results of three patients after 30 min are shown. Sample 1 contains cat allergen and water which was dialyzed against water and thus acts as a positive control. Sample 2 includes cat allergen and AS which was dialyzed against AS, thus the test sample contains AS. Sample 3 is cat allergen and AS which was dialyzed against water, therefore the test sample does not contain AS. Water and AS controls are also shown (samples 4 and 5 respectively). Representative photograph (patient 3) of skin test response after 30 min is also shown.

In order to be sure that AS was altering the allergen, and not modulating the hypersensitivity reaction by some other mechanism, mast cell stabilization for example, a simple experiment was performed. Cat allergen was mixed with AS (as described in *Methods *section), dialyzed against water to remove the AS, and then used for skin testing in several sensitized patients. Results shown in Figure 4 clearly demonstrate that AS reduced the allergen-induced skin prick response without being present in the test material. The reduction in wheal was identical between the sample containing AS and the sample which contained no AS, only exposed to AS and dialyzed to water. The experiment was repeated using Centricon ultrafiltration devises (Millipore, 3K MW cutoff) to remove AS from the samples with exactly the same results (data not shown). This indicates that exposing the allergen to AS results in alteration of its ability to induce a hypersensitivity reaction in sensitized patients.

## Discussion

Approximately 16% of persons living in the United States demonstrate an exaggerated tendency to mount IgE mediated response to a wide variety of common environmental allergens, leading to an estimated 1 of every 9 doctor visits [21,22]. Consequently, treatment modalities and applications that would prevent or significantly reduce exposure to such allergens are of considerable interest and importance [23]. In the past, attempts have been made by some investigators to reduce exposure by using certain chemicals known to bind to proteins [6-12]. For example, tannic acid and sodium hypochloride have been found to interact and form complexes with allergen-proteins [6-12]. Unfortunately, their use has been greatly limited due to staining and bleaching properties respectively. In this study, we examined and found that AS could be used to significantly reduce type 1 hypersensitivity reactions to several different allergens. Wheal and flare reactions to cat, dog, mite, cockroach, and Alternaria allergens were markedly inhibited by AS (Table 1, Fig. 1 &2). The mechanism(s) by which AS prevents allergens from interacting with their antigen-binding site on specific IgE molecules, bound to receptors on mast cells, remains unclear. One might however, envision the formation of ionic allergen-AS complexes or alteration of the allergen's protein structure by AS. Our experiments have demonstrated that exposing the allergen to AS results in this effect; AS does not have to be included in the test material to get the reduction in skin test response (Fig. 4). In addition, preliminary ELISA results show a reduction in allergen binding to specific monoclonal antibodies (data not shown). Several patients' (patients #16 & 26 to cat and #27 to mite) skin test results, however, did not appear to diminish with AS treatment of the allergen. These patients' responses are likely artificial, since they represent only 3 of 83 test results. An alternative explanation could be that the AS-altered allergen retains some epitopes which can be recognized by a very small percentage of individuals. Studies are currently underway to further elucidate the mechanism(s) by which AS interferes with this important interaction.

Aluminum sulfate does not appear to block histamine-induced responses. Skin reactions were not influenced by mixing AS with histamine or by injecting histamine into sites previously injected with AS (Fig. 3). Its effects on other mediators such as leukotrienes or proinflammatory cytokines remain to be determined. Finally, it should be noted that AS exhibits several properties that make it a great candidate for environmental control of allergens. It acts quickly; skin testing performed within 15 minutes of adding AS to the allergen exhibits a diminished skin test response. Allergen-AS mixtures are very stable, mixtures stored for up to 3 months produced a similar reduced skin reaction as those freshly prepared. Also it appears to lack any detrimental toxicity as determined by *in vitro *testing with cultured human endothelial cells and as indicated by the lack of any significant skin reactivity in patients above controls when injected alone. AS is inexpensive and does not appear to stain or discolor carpeting or clothing. Although our studies were done in the clinic, we feel that similar inhibitory effects (blocking of allergic reactions) will result from AS application to the environment. Active investigation is currently underway to evaluate the effectiveness AS have in inactivating allergens in the environment.

## Conclusion

AS was found to significantly reduce the skin test response in sensitized patients to each of the allergens tested: dust mite, Alternaria, dog, cat, and cockroach. The exact mechanism in which AS produces this effect is not known. However, our results demonstrate that AS does not block the effects of histamine, and produces its effect without being present in the skin test sample. It appears that AS alters the allergen, changing its epitopes, thus reducing the ability of specific IgE to bind and ultimately mast cell degranulation. AS does not stain, is cheap, nontoxic, is stable in solution, and appears to have long acting affects, making it a great candidate for use as an environmental control agent.

## List of abbreviations

AS, Aluminum sulfate; ID, intradermal; STR, skin test response

## Competing interests

The author(s) declare that they have no competing interests.

## Authors' contributions

CSS performed the patient testing, along with the clinical study design. SAS, TJG, and DEJ performed the laboratory testing and study design. All of the authors contributed to manuscript preparation.
